# Editorial: Peptidomimetics: Synthetic Tools for Drug Discovery and Development

**DOI:** 10.3389/fchem.2021.802120

**Published:** 2021-11-12

**Authors:** Annarita Del Gatto, Steven L. Cobb, Jinqiang Zhang, Laura Zaccaro

**Affiliations:** ^1^ Institute of Biostructures and Bioimaging, CNR, Naples, Italy; ^2^ CIRPeB, University of Naples “Federico II”, Naples, Italy; ^3^ Department of Chemistry, Durham University, Durham, United Kingdom; ^4^ School of Pharmaceutical Sciences, Chongqing University, Chongqing, China

**Keywords:** peptidomimetics, design, chemical synthesis, structure-activity relationship, drug discovery, drug development

The prominent role of peptides in controlling important physiological events and in influencing many pathological mechanisms is widely recognized. The chemical diversity of peptides is greater than that of any other class of biological molecules, and the conformational behavior is responsible for high activity, specificity, and affinity, features that are exploited to discover new promising molecules, some of which are currently in clinical trials or have already been approved as drugs (Del Gatto et al., 2021; Fosgerau and Hoffman, 2015). Nevertheless, many peptides do not enter clinical trials because of inherent challanges, such as enzymatic susceptibility and membrane impermeability.

Peptidomimetics are peptide analogs able to mimic the structural elements and functionality of natural peptides retaining the capability to interact with the biological target and produce the same biological effect.

The skill of transforming peptides into peptidomimetics is a growing approach in medicinal chemistry dedicated to overcoming the peptide drawbacks preserving the function and providing molecules poor in side effects and more active and selective.

The presented Research Topic includes papers and reviews, and it aims to provide an overview of the current state-of-the-art and recent advances within the field of peptidomimetics.

Herlan et al. explore cyclic peptoid-peptide hybrids as a new class of highly efficient, versatile transporters into mitochondria selecting the fluorophore Rhodamine B as cargo. The study performed on different cyclic tetramers and hexamers, containing several alkylated glycine monomers and up to two amino acids, indicates that the mitochondrial targeting of the hydrophobic transporters occurs without the need for additional cation charges residues. These data open the door for future investigations focused on a widespread comprehension of the structure-activity relationship that will increase the applicability of these systems in biomedicine as weapons for the treatment of pathologies associated with mitochondria.

The review by Staśkiewicz and colleagues provides an overview of the recent advancements in triazolyl-containing peptidomimetics. The authors highlight the most efficient synthetic methods for obtaining peptides containing 1, 2, 3-triazole moieties and report the promising activity of some triazolyl peptidomimetics as inhibitors of enzymes or as agents against cancer, viruses, bacteria, or fungi.

Allosteric modulators can display fewer side effects, improved selectivity, and enhanced biological specificity compared to orthosteric ligands of GPCRs. Given the aforementioned properties, allosteric modulators of GPCRs have emerged as promising therapeutic leads for a range of diseases. Despite the fact that many peptides (and proteins) have evolved to function as allosteric modulators, they remain largely unexplored as new leads for allosteric modulation of GPCRs. The review by Olson et al. highlights the sources and strategies that have been used to identify both peptide and peptidomimetic-derived allosteric modulators of GPCRs. It provides an overview of the approaches used to convert peptide leads into peptidomimetics. A critical discussion on the potential opportunities and challenges regarding the future development of peptidomimetics in the area of allosteric modulator drug discovery is also provided.

Interleukin-1β (IL-1β) binds to the IL-1 receptor (IL-1R) and in doing so it activates numerous downstream signaling mediators, including NF-κB, which is essential for cellular protection. The development of selective IL-1-targeting therapeutics has thus become a significant area of research activity. Geranurimi et al. describe the synthesis and biological evaluation of a series of modified peptides, which are analogs of the all d-amino acid heptapeptide rytvela, previously reported as an allosteric IL-1R modulator that conserves NF-κB signalling while inhibiting other IL-1-activated pathways. Key to the synthetic peptide work described is the effective solid-phase synthesis of (3*R*,4*S*)-β-substituted-α-amino-γ-lactam (Agl) peptides employing N-Fmoc protected Agl-dipeptide building blocks. The relevance of the β-substituent is evidenced in rodent models of preterm labor and retinopathy of prematurity, where the analog peptides prepared are found to inhibit preterm birth and vaso-obliteration, with equal and better activity relative to parent peptide. The structure-activity relationships of the analogs offer insight into the requirements for pharmacological selectivity of IL-1R modulators.

Antimicrobial peptides (AMPs) are recognized to be a precious source of potential drug leads as they prevent microbial resistance to conventional antibiotics. Thus, investigation of AMP interactions with animal cells and multicellular organisms is fundamental for their development as antibacterial drugs for humans, but only a few tools are currently available for such studies. In this context, Afonin et al. report the synthesis, biophysical, and *in vivo* studies of a known AMP, explored using a 3-hydroxychromone-derived fluorescent amino acid in place of the original aromatic amino acids. They evaluate the effects of such modifications in terms of structure-activity relationship and demonstrate that the use of this kind of peptidomimetics is an efficient strategy enabling *in vivo* peptide-cell and peptide-organism interactions monitoring.

In addition to functioning as antibacterial agents, AMPs have broad bioactivity, including anti-inflammation, antitumor, and antiviral. Peptide stapling strategies have been widely utilized to improve the pharmacological properties of peptides. All-hydrocarbon stapled peptides have attracted great attention due to their increased target binding affinity and enhanced protease stability. In this issue, Hu et al. design and synthesize a series of all-hydrocarbon stapled analogs of antimicrobial peptide A4K14-Citropin 1.1. Among the synthetic analogs, two stapled peptides display improved protease stability and increased antitumor activity compared to the flexible linear counterpart, which proves the all-hydrocarbon stapling technique is promising for the development of novel AMPs-based cancer therapeutics.

The collective manuscripts highlight recent progress in the field of peptidomimetics that represent an open door for the development of peptide-based therapeutics.

The synthetic tools introduced in the peptide molecules to enhance drug delivery, proteolytic stability, antimicrobial, and antitumoral activities as well as allosteric modulation, make peptidomimetic drug candidates highly attractive for development in disease outbreak situations.
